# Exploring cardiometabolic markers in adverse pregnancy outcomes: insights from the GROWell study

**DOI:** 10.21203/rs.3.rs-8844977/v1

**Published:** 2026-04-23

**Authors:** George E. Kuodza, Victoria F. Keeton, Logan A. Williams, Ray Kawai, Aron Judd P. Mendiola, Christina G. Torres, Paige M. Smith, Jennifer E. Phipps, Sebastian Castro-Alvarez, Paige D. Gilliland, Maressa L. Rodriguez, Kathryn A. Carbajal, Isabella C. Vo, Alina Patrikeyeva, Janine M. LaSalle, Leigh Ann Simmons

**Affiliations:** University of California; University of California; University of California; University of California; University of California; University of California; University of California; University of California; University of California; University of California; University of California; University of California; University of California; University of California; University of California; University of California

**Keywords:** Obesity, Adverse Pregnancy outcomes, Lipidomics, Cardiometabolic health, Dried blood spots

## Abstract

Women with pre-pregnancy overweight or obesity are at increased risk of adverse pregnancy outcomes (APOs) and postpartum weight retention (PPWR). We examined which lipid classes were associated with APO and PPWR during pregnancy and postpartum using a subsample from a clinical trial. Data were collected via questionnaires, electronic health records, and participant-collected dried blood spots at three time-points. Lipidomic profiles were measured at all three time points in 49 participants. Using weighted-lipid correlation network analysis, differential lipid analysis, and partial-least squares discriminant analysis, we identified triglyceride (TG)-rich lipid signatures associated with APOs and PPWR. In early pregnancy, three TG networks and seven individual TGs were consistently associated with APOs. Postpartum, several TG networks and individual TGs were associated with APOs and PPWR. These findings highlight TG lipids’ crucial role in pregnancy outcomes and the potential of TG-based lipidomic biomarkers for early risk identification to improve maternal and fetal health.

## 1. Introduction

Adverse pregnancy outcomes (APOs), such as hypertensive disorders of pregnancy, increase women’s long-term risk for cardiometabolic disease and mortality^1^ and negatively impact offspring^2^. Dysregulation of lipid metabolism at preconception^3^, during pregnancy^4–6^, and postpartum^3,7^ is associated with APOs, and lipids may serve as perinatal biomarkers of metabolic disease risk^5,8,9^. Dyslipidemia in the postpartum period is associated with postpartum weight retention (PPWR), which negatively affects future cardiometabolic health outcomes for women and is a risk factor for APOs in subsequent pregnancies^10–12^.

Alterations in traditional lipid panels have long been broadly associated with APOs and PPWR, with hypertriglyceridemia being the most predictive of adverse outcomes^4,10,13–16^. However, lipids include hundreds of species that vary in composition and function, grouped into categories such as triacylglycerols, ceramides, and glycerophospholipids^17,18^. Recent advances in lipidomic analysis have enabled more in-depth examinations of lipid metabolites, aiming to identify precise biomarkers of risk for APOs or future cardiometabolic abnormalities^3,5,9^. Some studies show associations between certain lipid species and gestational diabetes^3,5,7,9^, hypertensive disorders of pregnancy^6,8,19^, and PPWR^3^. However, these investigations vary in the timing of data collection, the APOs examined, and the analysis performed, making comparison across studies challenging. There is a critical gap in research that examines the dynamics of lipidomic profiles in women with pre-pregnancy overweight or obesity from early pregnancy through the early postpartum period and how these profiles relate to APOs using comprehensive analytic methods to validate findings.

The purpose of this study was to build on existing evidence linking lipidomic profiles to APOs and PPWR, and to identify groups of lipids early in pregnancy that could be predictive of APOs in a remote study of women with pre-pregnancy overweight or obesity. Specifically, the study aims were to: 1) perform untargeted lipidomic network analysis on biospecimens collected from women at 10 to 16 and 36 to 38 weeks’ gestation, and 3 months postpartum; and 2) test the associations between these lipid profiles, APOs, and PPWR. Findings may lay the groundwork for identifying biomarkers for future APOs and cardiovascular risk, which can be used for risk stratification and personalization of peripartum prevention and treatment of APOs.

## 2. Results

### Sample characteristics

Table 1 presents the sociodemographic and clinical data for the 49 participants. Median participant age was 34.54 years, and most participants identified as white (63.27%) or Hispanic (22.45%). Approximately 57% of the sample had a college degree or higher. Median pre-pregnancy BMI was 30, and the mean REAP diet quality score was 53.46. About one-third of participants experienced APOs, with the most common being preterm or early-term birth (16.33%) and hypertensive disorders of pregnancy (12.24%). The mean overall participant PPWR was 1.67 kg; in participants with excessive PPWR, the mean retained weight was 8.83 kg.

**Figure 1:**
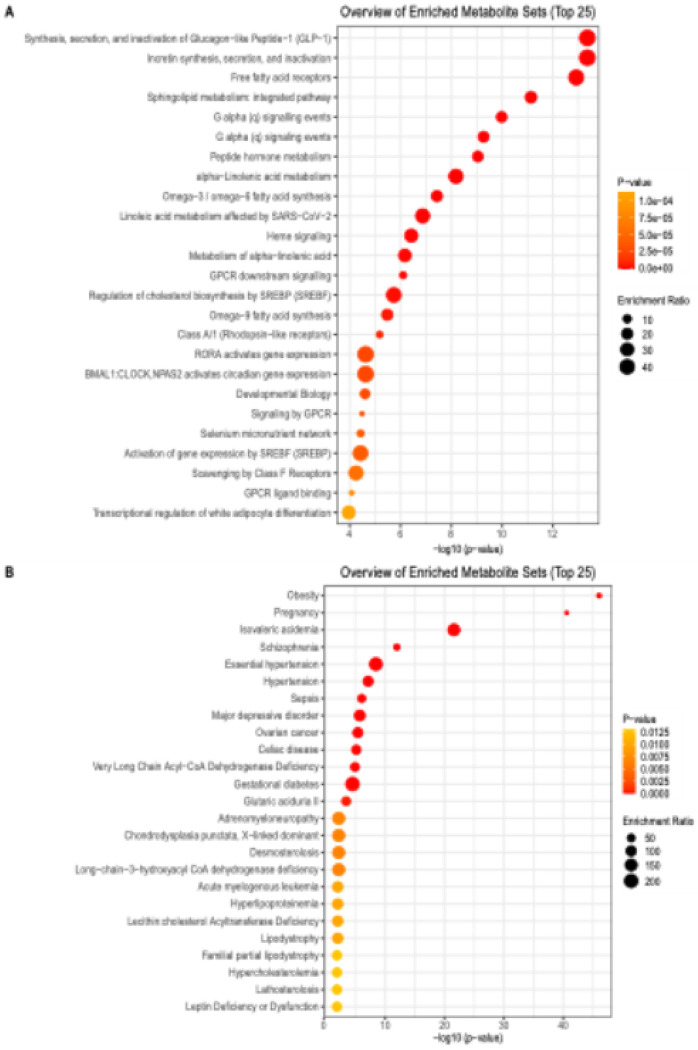
Enrichment Pathways **A.** Molecular pathways **B.** Disease-based pathways

Using untargeted lipidomic profiling, 451 annotated lipids were identified. Their lipid species included triglycerides (TGs), ceramides, fatty acids, sphingomyelins, and others (Table S1). Enrichment analysis of these 451 lipids identified lipid metabolism pathways, such as free fatty acid receptors, and omega-3/−6 fatty acid synthesis (Fig. 1A). Additionally, pathways related to glucose metabolism, such as synthesis, activation, and inactivation of GLP-1, were identified. For the disease-based pathways, obesity and pregnancy were the top main terms that were identified (Fig. 1B). Other pathways included an enrichment for pathways related to essential hypertension, hypertension, and gestational diabetes.

**Figure 2:**
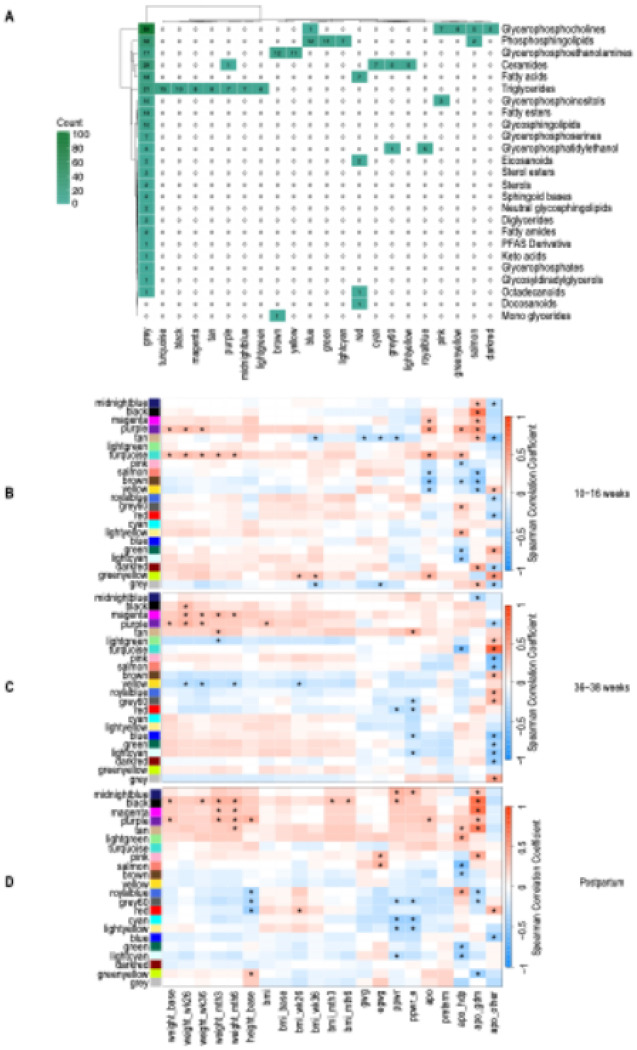
Weighted Lipid Correlation Network Analysis (WLCNA). **A.**Lipid class distribution. There were seven triglyceride (TG ) modules (turquoise, black, magenta, tan, purple, midnightblue, lightgreen ), two glycerophosphoethanolamine modules (brown, yellow), three phosphosphingolipid modules (blue, green, lightcyan), one fatty acid module (red), three ceramide modules (cyan, grey60, lightyellow), one glycerophosphatidylethanol module (royalblue), and four glycerophosphocholine modules (pink, greenyellow, salmon, and darkred). **B.** Module trait correlations at 10-16 weeks **C.** Module trait correlations at 36-38 weeks **D.**Module trait correlations at Postpartum

Network analysis of these lipids revealed 21 “consensus” modules that were used to compare the same groups of correlated lipids across different timepoints (Figure S2). Modules varied in group size (5–15 lipids), and the remaining 270 unassigned lipids formed a residual “grey” module. (Fig. 2A, Table S1). Among these were seven TG modules, two glycerophosphoethanolamine modules, three phosphosphingolipid modules, one fatty acid module, three ceramide modules, one glycerophosphatidylethanol module, and four glycerophosphocholine modules. Although some modules included more than one lipid species, most modules included a distinct group of lipids. Hierarchical clustering dendrograms (Figure S3-5) and heatmaps show the overall similarity of the consensus module networks (Figure S6).

### Outcome 1: APOs

Across three different approaches to identifying lipid predictive of APOs, TGs were positively associated with APOs in the 10–16-week timepoint. Network analysis revealed three TG modules that significantly positively correlated with APOs (strongest module r = 0.48, p = 0.000392) (Fig. 2B, Table S2). Additionally, subclassifications of APOs, such as having hypertensive disorders and gestational diabetes, also showed some positive correlations with five TG modules. On the other hand, three modules related to phosphatidylethanolamines and phosphatidylcholines were negatively correlated with APOs (Fig. 2B, Table S2).

**Figure 3:**
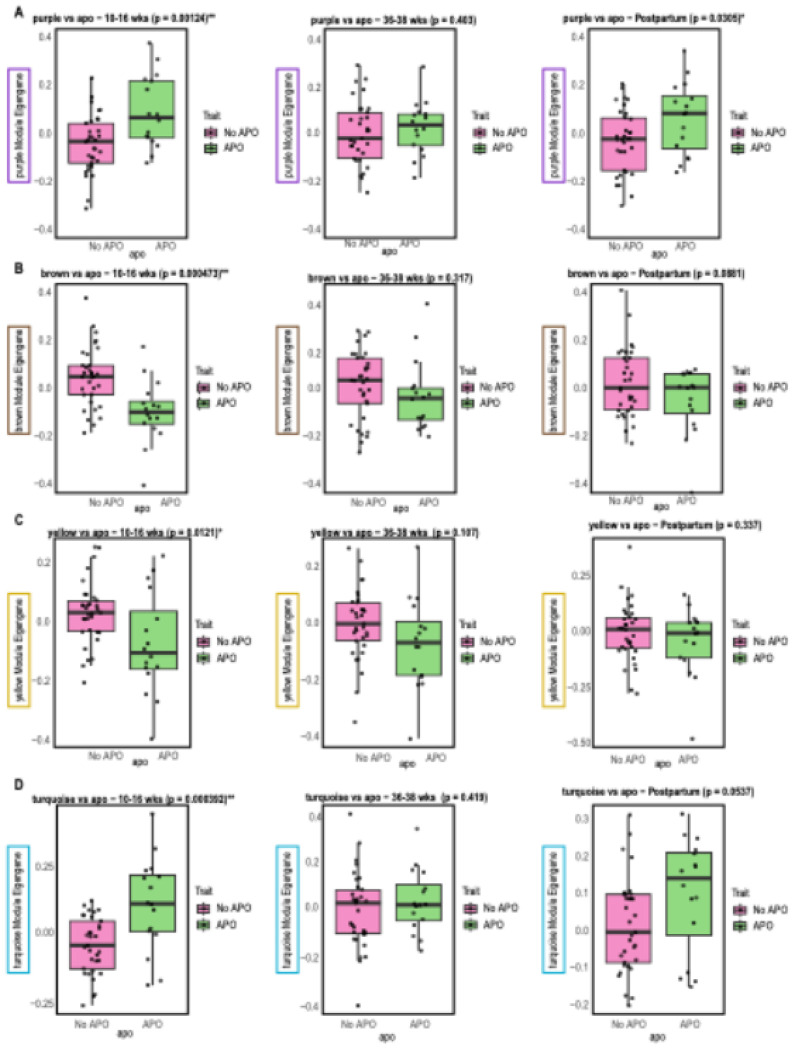
Module eigennode and APO plots. Each plot contains the module eigennode plotted against APO for each time point. **A.** Purple Module **B.**Brown Module **C.** Yellow Module **D.** Turquoise Module . The purple module showed strong associations at the 10-16-week and postpartum and the hub lipid was TG (50:2) for both timepoints. The other modules showed strong relationships at the 10-16-week time point and the hub lipids of the turquoise, yellow, and brown modules were TG (46:0), phosphatidylethanolamines (36:2), and phosphatidylethanolamines (P-36:4/O-36:5), respectively.

Next, we plotted the module eigennode against APOs for only four modules with significant APO associations at the 10–16-week timepoint. In the plots, most of these associations were specific to the earliest time point (Fig. 3, Table S3-5). The purple module was the only module that showed significant correlations with APOs at both 10–16 weeks and postpartum.

**Figure 4:**
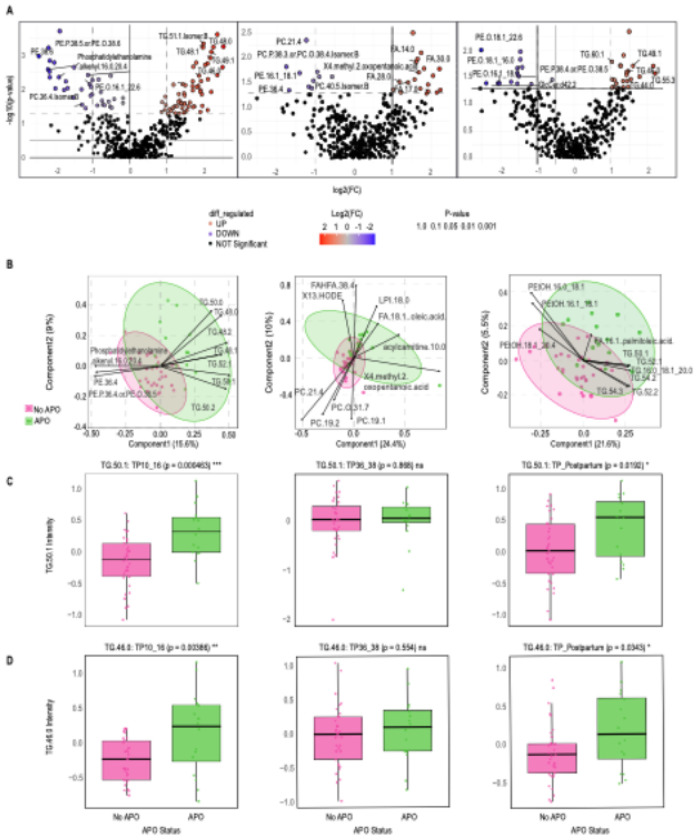
Changes of lipids for APOs vs no APOs across time points. **A.** Volcano plots show differential lipid regulation. The x-axis represents the log_2_ fold change (FC) between the two groups, while the y-axis plots the −log_10_(p) from the t-test of differences between samples, with red representing upregulation and blue representing downregulation. **B.**PLS-DA biplot showing top 10 lipids involved in separating APOs (green) and no APOs (red). **C-D.** Changes of the TG lipids **C.** TG (50:1) and **D.**TG (46:0).

Alternative approaches of differential lipid analysis and PLS-DA confirmed the results of the network analyses. Specifically, 78 differential lipids were differential by APO status at 10–16 weeks including a total of 31 TGs that were upregulated and positively associated with APOs, while 24 lipids were downregulated and enriched for phosphatidylethanolamines (Fig. 4A, Table S6). In the PLS-DA, among the top 10 lipids, seven were TGs positively associated with APOs, while the other three were phosphatidylethanolamines and were negatively associated with APOs (Fig. 4B). Overlapping lipids across methods included seven TGs that were positively associated with APOs, i.e., (48:0), TG (48:1), TG (48:2), TG (50:0), TG (50:1), TG (50:2) and TG (52:1), TG (48:0), TG (50:0), TG (50:1), TG (48:1), TG (52:1), TG (48:2), TG (50:2).

In contrast to the 10–16-week timepoint, none of the positive associations between TG modules and weight-related variables in the 36–38-week network analyses were associated with APOs (Fig. 2C, Table S7-8). Similarly, the differential lipid and PLS-DA analyses revealed that fatty acids, not TGs, were mainly positively associated with APOs (Fig. 4A,4B).

Overall, the strongest associations of TGs and APOs were seen at the early pregnancy and postpartum time points. While only one TG module was positively associated with any APOs (purple, r = 0.309, p = 0.0305) in the postpartum timepoint (Fig. 2D, Table S9), six TG modules were associated with hypertensive disorders and gestational diabetes in the postpartum period (strongest module, r = 0.703, p = 3.21^E-09^) (Fig. 1D, Table S9). TG (50:1) (Fig. 3C) and TG (52:1) were both altered at the 10-16-week time point and postpartum time points, PLS-DA (Fig. 4B). These two lipids were also part of the TG (purple) module, showing similar findings across methods. Additionally, some altered TGs from the postpartum differential lipid analysis (Fig. 4A, Table S10) were also altered at the 10-16-week time point, such as TG (49:1) and TG (46:0) (Fig. 4D).

### Outcome 2: PPWR

In contrast to lipid associations with APOs that predominated in early pregnancy, the PPWR outcome was predominantly associated with lipid profiles in the late pregnancy and postpartum period. In the network analysis, a TG module that had been negatively associated with total PPWR at the first timepoint was positively associated with excess PPWR at 36–38 weeks (tan, r = 0.363, p = 0.00992) (Fig. 2C, Table S7). At postpartum, two TG modules were positively associated with total PPWR. One TG module was positively correlated with both total and excess PPWR (midnightblue, r = 0.349, p = 0.0134) (Fig. 2D, Table S9).

**Figure 5:**
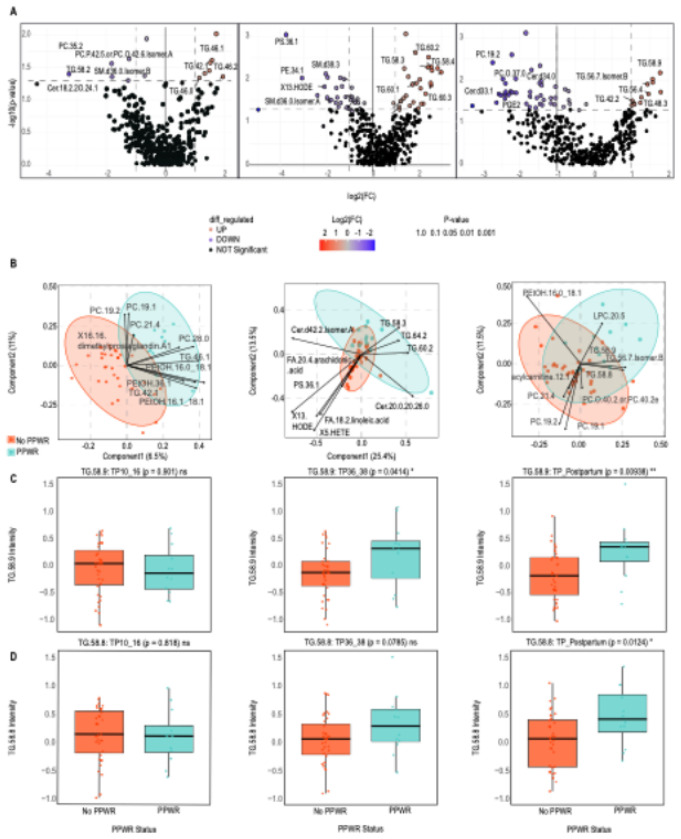
Changes of lipids for PPWR vs no PPWR across time points. **A.** Volcano plots show differential lipid regulation. The x-axis represents the log_2_ fold change (FC) between the two groups, while the y-axis plots the −log_10_(p) from the t-test of differences between samples, with red representing upregulation and blue representing downregulation. **B.**PLS-DA biplot showing the top 10 lipids involved in separating PPWR(green) and no PPWR (red). **C-D.** Changes of the TG lipids **C.** TG (58:9) and **D.**TG (58:8).

Early pregnancy differential lipid analysis revealed four TGs associated with PPWR (Fig. 4A, Table S11). In late pregnancy, among the five TGs associated with PPWR, four were found in the network analysis, which were TG (60:2), TG (60:3), TG (58:3), and TG (60:1) (Fig. 4A, Table S12). At postpartum, among the 10 upregulated lipids were seven TGs associated with PPWR (Fig. 5A, Table S13). Some were also found in the WLCNA modules like TG (58:9) (Fig. 5C), TG (56:7 Isomer B), TG (58:8) (Fig. 5D), and TG (56:8 Isomer A).

The late pregnancy PLS-DA identified three TGs positively associated with excess PPWR, which were TG (60:2), TG (58:3), and TG (64:2). (Fig. 5B). Two TGs were found in all three analyses at late pregnancy, TG (60:2) and TG (58:3). Postpartum PLS-DA revealed three TGs positively associated with excess PPWR (Fig. 5B). TG (58:9) and TG (58:8) (Fig. 5C–D) were in all three analyses at postpartum. Overall, TGs show strong signatures with PPWR in the late pregnancy and postpartum timepoint.

## 3. Discussion

To the best of our knowledge, this is the first longitudinal study to use participant self-collected dried blood spots in a remote setting to identify lipidomic trends in women entering pregnancy with overweight or obesity. Our combination of three complementary analytic methods produced a robust, comprehensive assessment that enabled us to showcase changes in different lipids and their associations with APOs and PPWR at three time points across the perinatal and postpartum periods.

We observed TGs that are significantly altered in the early pregnancy and postpartum time points and are positively associated with APOs. Additionally, we confirmed seven TGs (Table S14) in early pregnancy with positive associations across all three analytic methods, which also have been established as early pregnancy markers of APOs in previous studies: TG (48:0),^5,7^ TG (48:1),^5–7,9,30,31^ TG (48:2),^5–7,19^ TG (50:0),^5,30–32^ TG (50:1),^3,5,6,31^ TG (50:2),^3,5,19^ and TG (52:1),^3,5,7,30–32^. The convergences of the different analyses on the same TG species, and the reproducibility of these findings across multiple studies, strengthen their validity. This supports the hypothesis that maternal dyslipidemia early in gestation reflects metabolic risk and that these lipids can be used as early biomarkers of pregnancy complications.

We also noted positive correlations between APOs and TGs in the postpartum period, with TG (50:1) common in all three analyses (Table S15), which indicates that APOs result in altered lipidomic profiles that persist postnatally. This may elucidate mechanistic pathways for increased risk of future cardiovascular disease in women who experience APOs^33–35^; however, more research is needed. Taken together, these findings highlight the potential for personalized lipidomic biomarkers of APOs that could inform interventions to mitigate risk both during and after pregnancy.

In association with PPWR, while TGs at all time points were significantly altered, the postpartum period showed the strongest associations of correlated lipid networks. The PPWR-associated TGs at the early pregnancy time point differed from those observed in the late pregnancy and postpartum time points, which may reflect naturally evolving metabolic processes in the perinatal period^3,4^. Two triglyceride modules that were positively associated with PPWR at postpartum were also correlated with gestational diabetes, which supports other research linking the two and highlights the potential role of lipidomics in pathways between PPWR and known risks for gestational diabetes in future pregnancies^36,37^. Perinatal lipidomic profiles may be instrumental in the prevention of PPWR and interventions to promote future health after pregnancy. For example, two recent studies found that promotion of lactation in women with a history of gestational diabetes was found to downregulate glycerolipids (including TGs), and these profiles were associated with a lower risk of developing type 2 diabetes^38,39^.

In understanding the link between APOs and PPWR, the enriched pathways we identified were related to lipid and glucose metabolism, which are known altered biological processes in those experiencing APOs and PPWR. While measuring TGs by themselves might not pinpoint everyone who will develop APOs, these data could still play a useful role in prediction models that include other risk factors. Future research should focus on testing specific TG markers across different population groups to see how much they improve our ability to predict APO risk beyond what we already know from existing indicators.

We used three different methods for lipidomic analysis, which provided a more comprehensive examination and strengthened the validity of our findings that TGs are associated with APOs and PPWR. Another strength of our study is that the dried blood spots were self-collected by participants and sent to our lab via mail. This facilitated recruitment from a wider geographical area and improved retention, leading to a more inclusive sample of participants, which is very crucial for adequate representation in physiologic research.

Our findings should be interpreted in the context of two limitations. First, we did not account for participants’ food intake in relation to the timing of their specimen collection, which could have influenced their lipid levels. Second, the small sample size limited the number of participants with APOs and PPWR, which may have led us to be underpowered to accurately assess statistical significance. However, the validity of our findings is strengthened by the fact that some of the trends we observed reflected similar results in other larger studies.

In conclusion, using advanced technology in lipidomics, we identified potential biomarkers that can be utilized in clinical practice, and these have been replicated in some studies focused on APOs in pregnant women. These findings underscore the significant role of lipids in pregnancy outcomes, particularly for individuals with overweight or obesity. There is an urgent need for predictive, replicable biomarkers to identify those at high risk of APOs, and lipidomic biomarkers represent promising tools for early identification and intervention to improve maternal and fetal health.

## 4. Methods and Materials

### Study design and participants

The GROWell (Goals for Reaching Optimal Wellness) study was a remote, blinded, randomized clinical trial evaluating a digital dietary intervention aimed at reducing gestational weight gain and postpartum weight retention among pregnant and postpartum women with overweight or obesity^20^. Data were collected via online questionnaires, electronic health records (EHR), and participant-collected dried blood spots (DBS) at 10–16 weeks, 36–38 weeks, and three months postpartum.

Frequency statistics were calculated to summarize sociodemographic and clinical characteristics of the sample. In addition to the primary outcomes, clinical characteristics included the participant’s pre-pregnancy body mass index (BMI) and average dietary quality, assessed using the Rapid Eating Assessment for Participants (REAP) score^21^.

### Ethics approval

This study was conducted in accordance with the Declaration of Helsinki, the U.S Common Rule, and the International Council for Harmonization Good Clinical Practice (ICH-GCP). The GROWell was registered on ClinicalTrials.gov (NCT02904473; registered 19 September 2016). The study protocol was approved by the University of California, Davis Institutional Review Board (1399548–18) under Federalwide Assurance (FWA00004557), and all patients provided informed written consent prior to participation.

### Outcomes of interest

Our outcomes of interest were APOs and PPWR. APOs were defined as a composite outcome if participants experienced any of the following: hypertensive disorders of pregnancy, early or preterm birth (37 weeks or less), gestational diabetes, or other clinically documented pregnancy complications. First, we compared those with APOs (n = 16) and without APOs (n = 33). Then, we compared those with PPWR (n = 12) and without excessive PPWR (n = 35). PPWR was treated as a continuous measure of total weight retained in kilograms, and a categorical variable for excessive PPWR defined as weight retention greater than 105% of preconception weight at 6 months postpartum^22^. The overall goal was to identify associations between lipid classes at the two antepartum and one postpartum time points and between each of the outcomes - APOs and PPWR.

### Biospecimen lipid profiling

Untargeted DBS lipidomics was performed at the West Coast Metabolomics Center at UC Davis using a standardized electrospray ionization quadrupole time of flight (ESI-QTOF) LC-MS/MS lipidomics platform. The lipid extraction and data acquisition processes and protocols are described in detail elsewhere^23^. Raw spectra were processed using MS-DIAL^24^, followed by data cleanup using MS-FLO^25^ and annotations using the LipidBlast library^26^. Peak intensities were used to estimate relative lipid abundance. These intensities were normalized to internal standards and adjusted for technical variation using the systematic error removal by random forest algorithm^27^. Only annotated lipids (n = 451) were included for the downstream analysis. Before performing the statistical analysis, the data were normalized using median, Pareto scaling, and log_10_ transformation in MetaboAnalyst (v6.0)^28^.

### Pathway and enrichment analysis

Enrichment analysis was performed using the metabolite set enrichment module in MetaboAnalyst (v6.0)^28^, with all annotated lipids to identify enriched molecular pathways and disease-related terms. For the molecular pathways, we specifically selected the *RaMP-DB*, which contains 3694 metabolites and lipid pathways (integrating KEGG via HMDB, Reactome, and WikiPathways). For the disease signatures, we selected the *blood-based signatures*, based on 480 metabolite sets reported in human blood.

### Weighted lipid correlation network analysis

We constructed correlation modules using weighted-lipid correlation network analysis (WLCNA), which is an adaptation of weighted gene co-expression analysis (WGCNA)^29^. WLCNA groups together correlated lipids into colored modules on a scale-free topology. Modules were found through hierarchical clustering and dynamic tree cutting. We used the “consensus” module analysis because we wanted to compare which modules are consistently shared between two or more networks, as described for WGCNA^29^. To construct scale-free networks, we selected a soft power threshold of 24 (Figure S1).

For each time point, we then correlated each lipid module’s eigennode with our variables of interest. The eigennode represents the entire lipid module as a single value, calculated as the first principal component of all the lipids within the co-expression module^29^. To further visualize the associations of the four selected modules, we plotted the module eigennode compared to APOs. Additionally, for each of the selected modules, we examined the hub lipid, which is the top lipid with the most connections with other lipids within each module based on eigennode connectivity. The top 10-ranked lipids in each module were determined, with the hub lipid ranked first.

### Differential lipid analysis

The differential lipid analysis was performed on MetaboAnalyst (v6.0)^28^, using the univariate (volcano plot) workflow. For each time point, relative lipid intensities were compared between outcomes. Lipids with an absolute fold change of ≥ 2 (|log_2_FC| ≥ 1) and p-value < 0.05 were considered significantly altered. The results were visualized on volcano plots.

### Partial least squares discriminant analysis (PLS-DA)

The Partial least squares discriminant analysis (PLS-DA) was performed on MetaboAnalyst (v6.0)^28^ using the univariate (PLS-DA) workflow. The goal was to identify predictors of sample separation in our outcomes. PLS-DA also provided additional information as to which lipids were the most important for sample separations based on variables of importance in projection score > 1. We then plotted biplots of the top-10 lipids positively and negatively associated with each outcome at each time point.

## Supplementary Material

This is a list of supplementary files associated with this preprint. Click to download.

• SupplementaryFigures.docx

• SupplementaryTables.xlsx

## Figures and Tables

**Table 1:** Characteristics of the sample (N = 49)

Characteristic	Value
Age in years – median (range)	34.54 (23.68, 39.48)
Race and ethnicity – n (%)	
Asian	3 (6.12)
Black	1 (2.04)
Hispanic	11 (22.45)
Multi-racial/other	3 (6.12)
White	31 (63.27)
Highest education level – n (%)	
High school/some college	21 (42.86)
Post-baccalaureate	28 (57.14)
Pre-pregnancy body mass index – median (range)	30.00 (25.00, 39.90)
Average diet quality score (REAP) – mean (SD)	53.46 (5.52)
Adverse pregnancy outcomes* – n (%)	16 (32.65)
Pre-term/early-term birth	8 (16.33)
Gestational diabetes mellitus	1 (2.04)
Hypertensive disorder of pregnancy	6 (12.24)
Other	3 (6.12)
Postpartum weight retention in kg – mean (SD)	1.67 (5.25)
Without excessive PPWR (n = 35)	−0.79 (2.94)
With excessive PPWR (n = 12)	8.83 (4.27)

* Total includes participants with more than one APOs

Overall lipid profiles

## Data Availability

The datasets generated and/or analysed during the current study are not publicly available as they have been deposited but are currently under review in the Metabolomics Workbench (DataTrack ID 6878), but are available from the corresponding author on reasonable request.

## Code Availability

The underlying code for this study is available on GitHub and can be accessed via this link https://github.com/dreusebio/lipid-consensus-wlcna-pipeline/tree/main/scripts_for_growell_lipidomics_analysis
